# P-642. Attributes of Point-of-Care Nucleic Acid Amplification Tests for Respiratory Syncytial Virus (RSV): Results of a Systematic Literature Review

**DOI:** 10.1093/ofid/ofaf695.855

**Published:** 2026-01-11

**Authors:** Kashmira Date, Kyle Fowler, Katherine Perez, Sima S Toussi, Mary Lynn Baniecki, Elaine Thomas, Katherine Schneider, Ornella Ruiz, Suzie Seabroke, Bradford D Gessner, Elizabeth Begier

**Affiliations:** Pfizer, Inc., Atlanta, GA; Pfizer, San Francisco, California; Pfizer, Inc., Atlanta, GA; Pfizer Inc., New York, New York; Pfizer Inc, Cambridge, Massachusetts; Pfizer, San Francisco, California; Pfizer, San Francisco, California; P-95, Barranquilla, Colombia, Atlantico, Colombia; P95, Leuven, Vlaams-Brabant, Belgium; EpiVac Consulting, Anchorage, Alaska; Pfizer Vaccines, Dublin, Dublin, Ireland

## Abstract

**Background:**

RSV disease burden in adults is often underestimated (in part due to lower viral loads undetected by low sensitivity antigen tests), infrequent standard-of-care testing and lack of robust home tests. Diagnostic confirmation relies on laboratory-based RT-PCR methods, requiring sample transport, processing, and ∼24-hour turnaround time for test results. Point-of-care (PoC) testing offers opportunities for faster (≤1 hour), close-to-patient care testing. We reviewed available literature to describe attributes of available PoC testing systems utilizing RT-PCR and other Nucleic Acid Amplification Tests (NAATs) for RSV detection.

**Figure:**
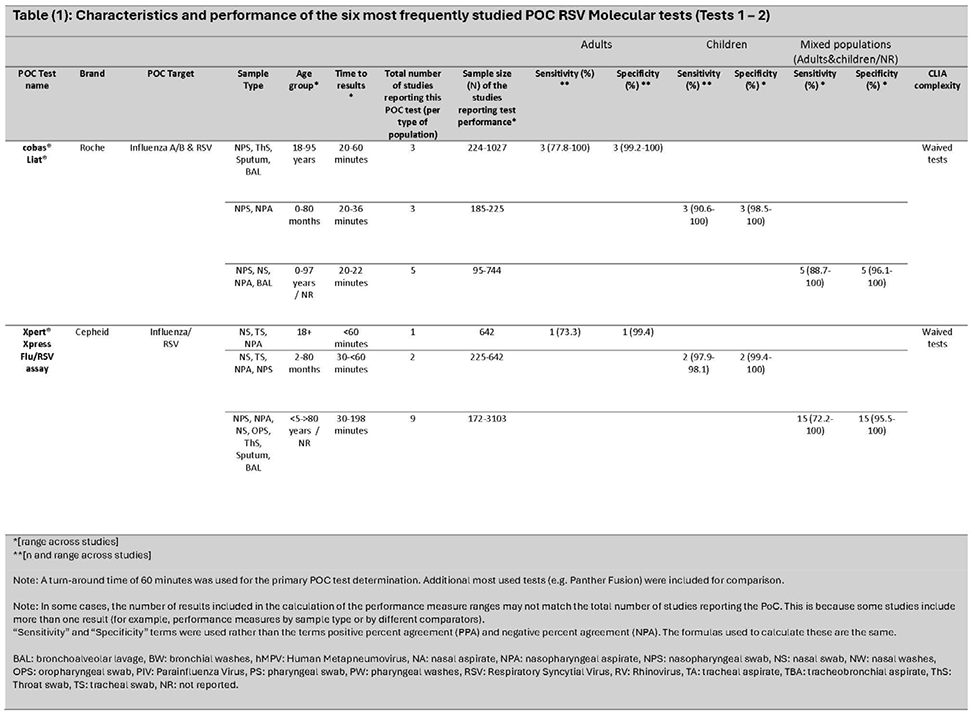

**Figure:**
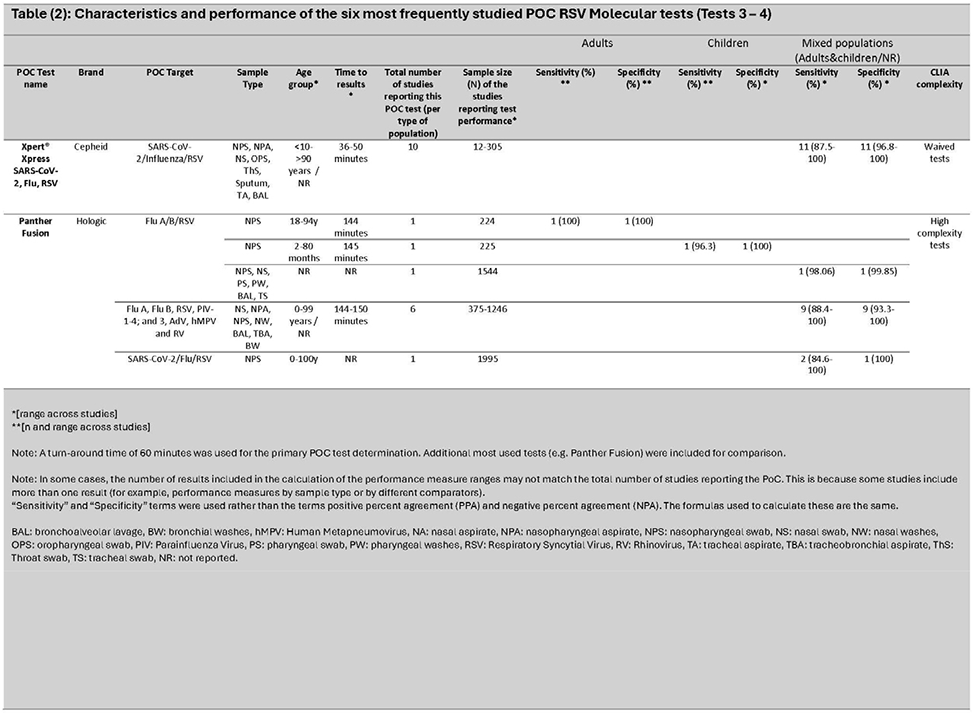

**Methods:**

We conducted a systematic literature review following the Preferred Reporting Items for Systematic Reviews and Meta-Analysis (PRISMA) guidelines, on PCR/NAAT-based PoC testing methods, both approved and under development during 2018–2024, and describe characteristics/performance and comparability with traditional RT-PCR methods.

**Figure:**
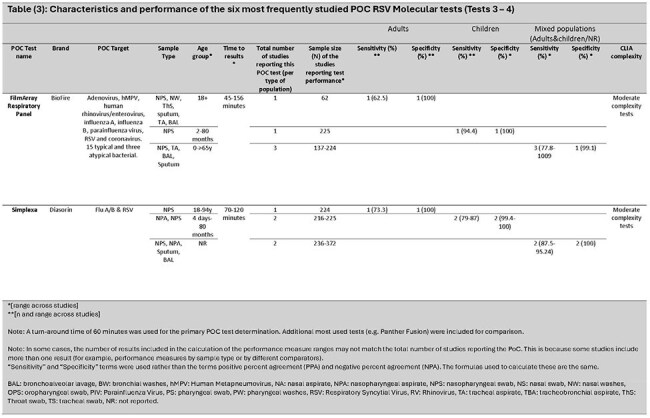

**Results:**

Of 3,915 articles identified, 75 met the inclusion criteria. Overall, 8% involved adults only ( >18 years), 17% children only (≤18 years), 36% mixed ages (children and adults), and 39% where age was not reported. Twenty-nine (39%) were conducted in the Western Pacific region, 22 (29%) in Europe, and 20 (27%) in the Americas. Most study samples (41; 55%) came from medical settings (inpatient and outpatient). Overall, 40 different PoC tests were identified (21 PCR, 19 other NAAT). CLIA complexity levels varied: low/waived (3), moderate (2), and high complexity (1). Six with the most available data are reviewed (*Tables 1 - 3)*. Compared with traditional RT-PCR, test sensitivity for children, adults, and mixed age populations ranged from 79–100% [Median (M): 96.3%]; 62.5–100% [M: 77.8%]; and 72.2–100% [M: 98.2%], and specificity ranged from 98.5–100% [M: 100%]; 99.2–100% [M: 100%]; and 93.3–100% [M: 100%], respectively.

**Conclusion:**

Current PoC molecular testing for RSV detection have acceptable turnaround times and high specificity. However, sensitivity was lower than traditional RT-PCR. Availability of reliable, cost-effective, scalable PoC molecular tests would allow rapid accurate RSV diagnosis to inform clinical management and facilitate clinical (e.g., antiviral) and epidemiologic research.

**Disclosures:**

Kashmira Date, MD. MPH, J&J: Prior employee|Pfizer, Inc.: employee|Pfizer, Inc.: Stocks/Bonds (Private Company) Kyle Fowler, PhD, Pfizer: Employee Katherine Perez, PhD, Pfizer: Stocks/Bonds (Public Company) Sima S. Toussi, MD, Pfizer: Stocks/Bonds (Private Company) Mary Lynn Baniecki, PhD, Pfizer, Inc: Salaried Employee|Pfizer, Inc: Stocks/Bonds (Public Company) Elaine Thomas, PhD, Pfizer: Stocks/Bonds (Public Company) Katherine Schneider, PhD, Pfizer: Stocks/Bonds (Public Company) Ornella Ruiz, MD, MSc, Pfizer Inc.: I am an employee of P-95, a company that was contracted by Pfizer to perform the systematic literature review (SLR) Suzie Seabroke, PhD MSc BSc, Pfizer Inc.: I am an employee of P-95, a company that was contracted by Pfizer to perform the systematic literature review (SLR) Bradford D. Gessner, MD, MPH, Pfizer: Stocks/Bonds (Public Company) Elizabeth Begier, MD, M.P.H., Pfizer: I am an employee.|Pfizer: Stocks/Bonds (Public Company)

